# Factors influencing the transition and retention of mental health nurses during the initial years of practice: Scoping review

**DOI:** 10.1111/jonm.13882

**Published:** 2022-11-23

**Authors:** Bindu Joseph, Sini Jacob, Louisa Lam, Muhammad Aziz Rahman

**Affiliations:** ^1^ Institute of Health and Wellbeing Federation University Berwick Victoria Australia; ^2^ School of Nursing and Midwifery Monash University Clayton Victoria Australia; ^3^ Graduate Studies, Institute of Health and Wellbeing Federation University Berwick Victoria Australia

**Keywords:** early‐career, factors, initial years, mental health nurses, retention, transition

## Abstract

**Aim:**

This review aims to identify the factors influencing the transition and retention of mental health nurses during the initial years of practice, recognize gaps in the literature and propose evidence‐based strategies.

**Background:**

Mental health is a challenging specialty; recruitment, transition and retention of mental health nurses are known issues of concern.

**Evaluation:**

The present study undertakes a scoping review to identify factors influencing the transition and retention of mental health nurses during the initial years of practice and the gaps in that research domain. A literature search was conducted using electronic databases. To gain an understanding of the topic of interest, the review of the literature extended from 2000 to 2022.

**Key issues:**

Existing evidence focuses on specific perspectives of transition. There is limited literature on factors influencing transition and retention among mental health nurses. Findings suggested that personal and professional factors could influence the transition and retention of mental health nurses during the initial years of practice. The main themes identified were personal attributes and professional factors with a number of subthemes.

**Conclusion:**

The scoping review identified only a few studies, which showed personal and professional factors related to the transition and retention of mental health nurses at the early stages of their career.

**Implications for nursing management:**

Potential benefits of effective transition and support with the understanding of factors influencing transition and retention of early career mental health nurses will enhance staff morale, sustainability of the workforce and better patient outcomes. Additionally, a few recommendations for nurse managers and leaders to improve transitional experiences and retention of early career nurses are highlighted.

## 1 INTRODUCTION

Mental health is a challenging specialty for various reasons (Joseph et al., 2022) such as the need for advanced training and qualifications, a volatile work environment, stigma toward mental illness, safety concerns and the need to establish a therapeutic relationship with clients with mental illness (Joubert & Bhagwan, 2018; The Productivity Commission, 2020). Mental health nurses are the largest workforce in mental health in Australia (Australian Institute of Health and Welfare [AIHW], 2020; World Health Organization [WHO], 2020) and many other countries. Recruitment, transition and retention of mental health nurses are a global concern. Several factors can affect the transition and retention of newly qualified nurses working in mental health. Furthermore, new graduate nurses transitioning to clinical practice face a high level of stress. They often feel overwhelmed by the challenges of the unfamiliar work environment. As they move from student nurses to registered nurses, their workload and responsibilities increase, expectations from the public, their managers and superiors from their organizations' increase, and organizational pressure to perform also increases along with specialty‐specific challenges (AIHW, 2020; WHO, 2020).

## 2 BACKGROUND

Transition is referred to as the process of changing from one condition to another along with roles and responsibilities (Meleis, 2010). Nurse retention is defined as the ability of an organization to keep its employees (Siddiqui & Jamil, 2015). A mental health nurse is a registered nurse who specializes in working with people with mental illness (AIHW, 2020). According to the Victorian Mental Health Wellbeing and Workforce Strategy (Department of Health, 2021), specialist skills for clinical staff in mental health require additional training after general nursing qualifications (Bachelor of Nursing). Graduate programmes in mental health can last for 1–2 years with or without postgraduate training. Concerns regarding the attrition of early career nurses were highlighted in the Australian context (Health Workforce Australia, 2012). Djukic et al. (2013) define early career nurses as nurses who practised nursing for less than 5 years. In this study ‘the initial years of practice’ refer to the first 3 years of practice in mental health nursing.

Nurse retention, particularly during the initial years of practice is a significant common problem across the world (Van Camp & Chappy, 2017). A study conducted in America showed that about 17.5% of new registered nurses leave their job within 1 year of starting jobs, with an additional one‐third leaving within 2 years. The average nurse turnover rate is 19.1%, and a nursing vacancy rate of 8% exists (Kovner et al., 2014). Another large study from Australia showed an average annual nurse turnover rate of 15.1% (Roche et al., 2015). Likewise, an international study also indicated that the attrition rates of new graduate nurses in their first year of practice range between 30% and 60% (Krugman et al., 2006; Goode et al., 2013). This has a heavy impact on the sustainability and skill mix of the workforce (Parker et al., 2014; Rush et al., 2013). Additional impacts of attrition could be emotional and financial struggles for nurses and financial implications and health care services (Goode et al., 2013).

Existing literature indicates a shortage of mental health nurses in Australia (Department of Health, 2021) with more shortages expected. Interestingly, 22.3% of current mental health nurses are aged 45 or above with 31.3% aged 55 and older (AIHW, 2020). This indicates a predictable shortage of mental health nurses in the next 10 years. At the same time, the World Health Organization also has predicted a worldwide shortage of nurses by 2030 (World Health Organization, 2020). Shortage and deficits of mental health nurses can affect health outcomes and the quality of care offered to patients with mental illness (Chiao et al., 2021). Additionally, the Australian College of Mental Health Nurses (ACMHN) submission in the Productivity Commission Inquiry (2020) pointed out that the Australian health care system predicted it would be unable to meet the mental health nursing demand without developing a mental health workforce. In the United Kingdom, there is an existing shortage of 40% of mental health nurses (Beech et al., 2019). A large survey (*n* = 498) of Victorian mental health nurses identified that young mental health nurses under 4 years of experience highlighted a high level of stress and lowered mental health (Foster et al., 2021) and suggested that new graduate mental health nurses need urgent support.

Nursing turnover within the first year varies between 5% and 60%, and literature from Australia suggests annual turnover between 12% and 38% (Hayes et al., 2012; Mills et al., 2016). The issues associated with retention within the early years of practice are consistently echoed in the literature (Government of Australia, 2020; Hayes et al., 2012; Mills et al., 2016). Low levels of retention and higher turnover in mental health than in general settings were already mentioned in the international context (Adams et al., 2021; Buchan et al., 2019). Furthermore, mental health care in rural and remote areas is covered by mental health nurses (National mental health workforce strategy, 2021–2031). Recruitment and retention are also challenging in this area (Australian Nursing and Midwifery Foundation, 2021).

In this context, recruitment, positive transition and retention are vital to ensure quality mental health services. Understanding the factors influencing the transition and retention of new mental health nurses is essential for a sustainable mental health workforce. There are several earlier studies completed in different countries on factors influencing the retention and transition of nurses during the initial years of practice in the general hospital setting and specific nursing specialties. However, only a few of those earlier studies exploring nurses' transition and retention topics were focused on mental health nurses.

## 3 METHODS

### 3.1 Design

A scoping review was conducted to map the research completed in this area and identify any existing knowledge gaps. This review method was adopted due to the nature of the topic and the flexibility to include a range of articles using various methodologies without the need for an individual quality appraisal (Pham et al., 2014). The information available in the literature about factors influencing transition experiences and retention of mental health nurses in the early stages of their careers was sought. The titles and abstracts of the papers were screened using Covidence software (Covidence systematic review software, n.d.). Two authors (S. J. and B. J.) screened the titles and abstracts of each paper and assessed them for relevance based on the inclusion and exclusion criteria. Irrelevant articles and duplicates were excluded. Studies were reviewed in full when identified as relevant based on the content of the abstract. In addition, the reference lists of pertinent papers were screened for additional articles. The study protocol was not registered. PRISMA‐ScR checklist was followed to ensure adherence to scoping review guidelines.

### 3.2 Search methods and inclusion/exclusion criteria

The key phenomenon of interest in this review was the factors influencing the transition of nurses during the initial years of practice in mental health. The PICo framework was used to develop the review question. The studies used for this review were identified based on the PICo criteria outlined: Population—mental health nurses; interest—factors influencing transition and retention; context—during the initial years of practice. Eligible studies included original peer‐reviewed research articles, relevant reviews on transition experiences, factors influencing transition, and retention to mental health nursing and published in the English language. The search extended to peer‐reviewed research articles published between 2000 and 2022 due to the significant advancement of mental health training and practices in the past 20 years. The exclusion criteria included articles not addressing factors influencing the transition and retention of mental health nurses during the initial years of practice or not exploring relevant experiences, or research articles focused only on general nurses.

The search strategy used was MeSH terminology and keywords, to identify all relevant studies on the topic. Then the Boolean operators ‘OR’ and ‘AND’ were used to combine results. The literature for this review was completed by searching the following electronic databases in 2021 and the last search was conducted in January 2022.Academic Search Complete, CINAHL Complete, MEDLINE, EBSCO, Pubmed, Web of Science and PsycINFO. A combination of search terms has been used. Key terms were searched individually and together using Boolean logic and proximity indicators for the various databases. As given ((MH ‘New Graduate Nurses’) OR (MH ‘Novice Nurses’) OR (‘graduate* N3 nurs*’) OR (‘novice* N3 nurs*’) OR (‘first‐year’ N3 nurs*) OR (‘newly employ*) OR (‘first‐year’ N3 practice) ((MH ‘Psychiatric Nursing’) OR (‘mental health’ N4 nurs*))(((‘Psychiatric Nurses’) OR (‘mental health’ Near/4 nurs*)))) AND TS = ((‘New Graduate Nurses’) OR (‘Novice Nurses’) OR (‘graduat* Near/3 nurs*’) OR (‘novice* Near/3 nurs*’) OR (‘first year’ Near/3 nurs*’) OR (‘newly employ*) OR (‘first year’ Near/3 practice)) (Retention) (staff retention), (nurse retention). ([MH ‘Nurses+’] OR nurse*) AND (transition* OR retention* OR [MH ‘Personnel Retention’]) AND ((initial N3 year*) OR (early N3 year*) OR (graduate* N3 year*) OR (new* N3 graduate*)).

## 4 FINDINGS

**FIGURE 1:**
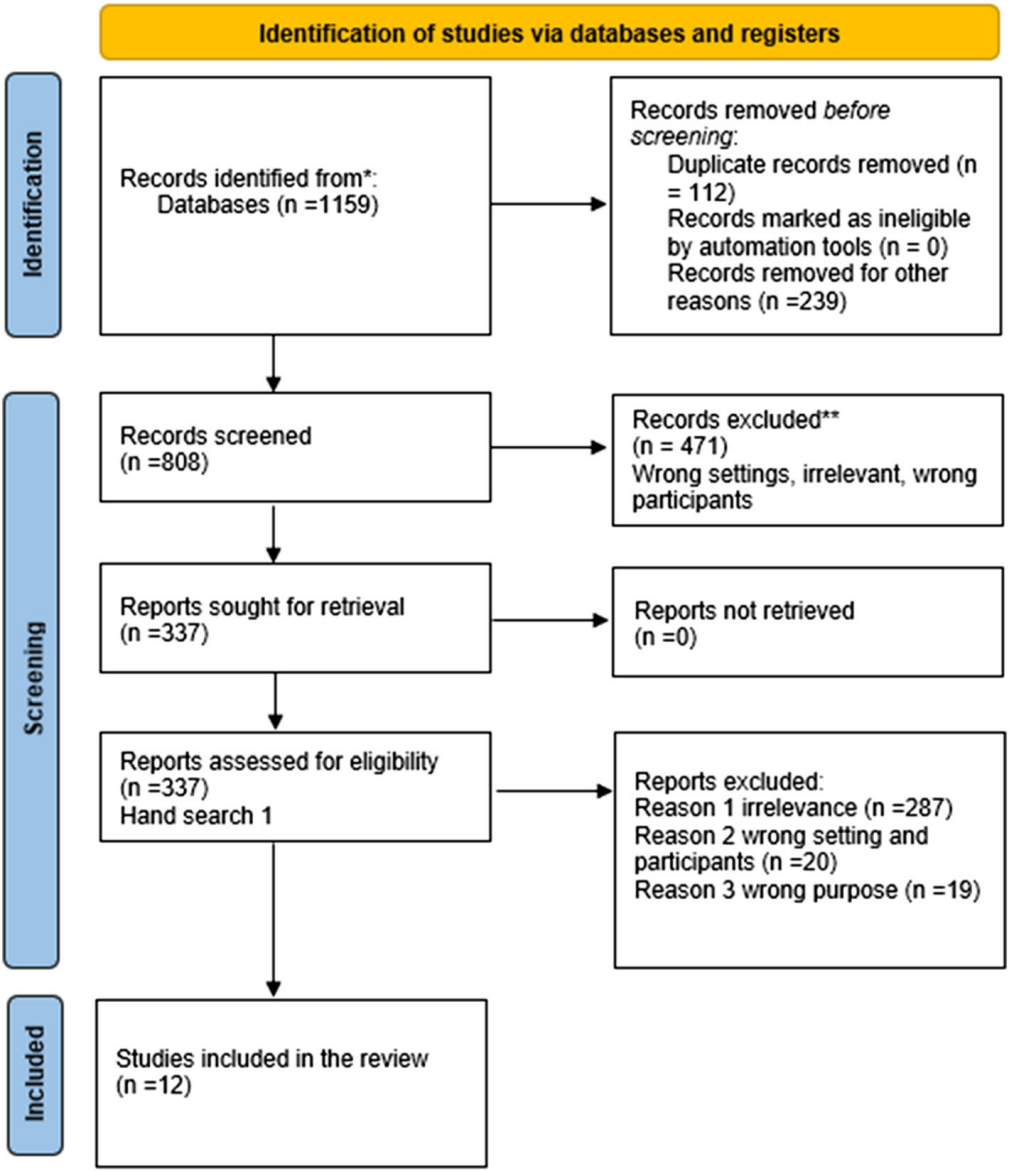
PRISMA flow chart

A total of 1159 studies were identified and out of those, 361 studies were removed mainly due to duplicates. Title and abstract reviews of 808 studies were conducted using Covidence software and 471 were excluded due to irrelevance. Full texts of 338 studies were retrieved and reviewed. Rigour was ensured through the independent review by two authors. The discrepancies were discussed, and the decision was made in agreement with the two authors. A hand search of the reference list was carried out to capture additional papers and yielded one additional study. Full texts were accessed if the abstract needed further clarification. Twelve studies that met the inclusion criteria and were relevant to the scoping review question were included in this review. Figure 1 shows the PRISMA flow chart of the search (McGowan et al., 2020; Page et al., 2021), and Table 1 demonstrates the details of the included articles.

**TABLE 1:** Details of selected literature

Author(s) year of publication	Study design/aim	Setting/sample	Methods	Key findings that relate to the scoping review question/s
Karlowicz & Ternus, 2009 United States	Grounded theory/aim: to explore the work experiences of psychiatric‐registered nurses (RNs) that influence retention within the first year of employment	*N* = 14 psychiatric nurses during the first year of employment with four psychiatric inpatient facilities	Interviews and case analysis	The new mental health nurses' issues involving education and training, team dynamics and organizational support converging to influence nurses' decisions to terminate employment within the first year (professional factors).
Hazelton et al., 2011 Australia	Participatory action research/aim: to evaluate a group mentorship programme for new graduate nurses working in an Australian public mental health service	*N* = 18 graduate nurses (within the first year of nursing) working in an Australian public mental health service	Group discussions, participatory observation	The new mental health nurses experienced difficulties in fitting into their work environments, neglectful and hostile treatment of patients, and the obstacles that hindered therapeutic skill acquisition (professional factors).
Schwartz et al., 2011 Canada	Qualitative study/aim: to explore the new nurses' experience of their role within interprofessional health care teams in a mental health organization.	*N* = 10 new nurses (within the first year of nursing) in a public mental health organization in Canada.	Semistructured interviews	The new mental health nurses found difficulties fitting into the new work environment. Establishing credibility and building trust were central to the new nurses' transition experiences. Interpersonal and organizational factors contributed to the transition (professional factors).
Wright et al., 2011 Canada	Qualitative study/aim: to identify the aspects of a successful integration experience into mental health nursing from the perspectives of new nurses.	*N* = 10 new mental health nurses (within 18 months of nursing) in a mental health setting in Canada.	Semistructured interviews	Results of this study stated that a quality relational connection between and among team members sustains the motivation to continue mental health nursing (personal and professional factors). A negative relational atmosphere sets the stage for new nurses to experience disillusionment and despair.
Hung et al., 2014 Taiwan	Qualitative study/aim: to understand the working experiences of new psychiatric nurses during their first year in a clinical setting.	*N* = 15 new psychiatric nurses in Taiwanese mental health services (within the first year of nursing)	Phenomenological approach semistructured interviews	The new mental health nurses experienced a lack of sense of security, challenges with learning the process of interaction with clients and families, learning an appropriate role in creating a therapeutic environment and belonging (personal and professional factors)
Young et al., 2018 South Korea	Qualitative study/aim: to describe the clinical experience of adaptation of new nurses working in psychiatry	*N* = 10 new psychiatric nurses (within the first year of nursing) in hospital South Korea	Giorgi's phenomenological method. In‐depth personal interviews	The new mental health nurses' experiences were themed as frustrations due to lack of nursing capacity, ‘heavy workload’, trying to find own place in the new workplace and developing own life (professional factors)
Mabala et al., 2019 South Africa	Qualitative descriptive design/aim: to explore and describe the adaptation process of newly qualified nurses working in public mental health facilities in South Africa.	*N* = 11 newly qualified nurses'(within 18 months of nursing) working at mental health facilities in South Africa	Unstructured individual interviews	Fear related to the mental health care environment; self‐doubt upon meeting the expectations of the inter‐professional team; ways to adjust to the challenges; and confidence as mental health care professionals (personal and professional factors)
Rahmani et al., 2019 Iran	Qualitative content analysis/aim: to examine the perception of the Iranian psychiatric nurses in psychiatric wards and their transition period.	*N* = 18 new Iranian psychiatric Nurses'(in the first year of mental health nursing) perception of transition in psychiatric wards	Unstructured interviews	The major themes in terms of transitioning were inadequate preparation, stress sense of self‐awareness and adjusting to the new workplace (personal and professional factors).
Pelletier et al., 2019 United States	Quantitative time‐sequenced comparative study Aim: to understand the effectiveness of a nurse residency programme in retaining new graduate nurses in a psychiatric–mental health setting	*N* = 34 new graduate nurses in a psychiatric–mental health setting (within two years of nursing)	Survey Quantitative time‐sequenced comparative study	The study yielded a turnover rate of 11.7% in Year 1 (88.3% retention) and 2.9% in Year 2 (97.1% retention rate), which are lower than the reported turnover rates (17.5% and 33.5%, respectively). Significant correlations are stated with knowledge and skills, social support, organizational support, civility, coping self‐efficacy, organizational and occupational commitment, person–organizational fit and burnout (personal and professional factors)
Kaihlanen et al., 2019 Finland	Quantitative This study aimed to examine the association between the final clinical practicum experience with transition experience and turnover intentions of newly graduated nurses	*N* = 712 Nurses graduated (within the first year of nursing) within the past 2 years	Survey with five subscales	The association between the clinical experience and turnover intentions (*β* = 0.23, *p* = .002) was partly mediated by the emotional distress and socio‐developmental‐role conflict and ambiguity (*β* = 0.26, *p* < .001) domains of the transition wand were associated with turnover intentions (personal and professional factors).
Cao et al., 2021 China	Quantitative This work aimed to examine the mediating role of transition shock on the relationships between resilience, social support, work environment and turnover intention in newly graduated nurses	*N* = 361 Chinese newly graduated nurses (in the first year of nursing)	Transition shock scale for newly graduated nurses Survey	The average levels of compassion satisfaction, burnout and traumatic stress in newly graduated nurses were 80.2%, 38.2% and 57.5%, respectively. Resilience, social support and work environment are directly related to transition shock and thereby retention (personal and professional factors)
Ho et al., 2021 United Kingdom	Aim: to explore how newly qualified nurses' work experiences are constructed through the interplay between self, workplace and home life influencing their retention	*N* = 46 (*n* = 12 new mental health nurses within 2 years of nursing)	Qualitative Semistructured interviews	‘Transition shock’, ‘workplace factors’ and ‘work/life balance’ have influenced the retention of nurses Eight participants had changed jobs or left, and two were looking to leave nursing (professional factors)

Most of the studies included in this review were qualitative (*n* = 9) and other studies (*n* = 3) were quantitative. Countries of origin were Australia (*n* = 1), Canada (*n* = 2), the USA (*n* = 2), Taiwan, Finland, South Africa, Iran, the United Kingdom, China and Korea (*n* = 1 from each country). The terms mental health nurses' and psychiatric nurses were interchangeably used in the literature for nurses caring for individuals with mental health disorders. ‘New mental health nurses’ and ‘graduate nurses’ were also used in the literature for nurses with less than 2 years of experience. All studies (*n* = 12) focused mainly on the transition experiences of new mental health nurses. An overview of the literature identified is portrayed in Table 1. Additionally, further review and analysis of identified literature elicited a few subthemes. The review elicited two main themes: personal factors and professional factors and six subthemes: sense of belongingness, self‐efficacy and self‐awareness, team dynamics and collegiality, workplace culture, mentorship and support and professional development.

### 4.1 Personal attributes

The influence of personal factors on the transition and retention of mental health nurses had been identified in the literature (Ho et al., 2021; Hung et al., 2014). In this literature review, some studies focused on the influences of personal factors on the transition experiences and retention of newly qualified nurses (Ho et al., 2021). Personal factors were mainly identified as the experience of belongingness, self‐efficacy and self‐confidence (Ho et al., 2021; Hung et al., 2014; Young et al., 2018).

#### 4.1.1 Perceived sense of belongingness and connectedness

A sense of belongingness in the work environment is an important element of the transition and retention of new nurses. Studies highlighted that these newly qualified nurses were trying to ‘find a place’ and ‘fit in’ to the workplace (Hazelton et al., 2011; Schwartz et al., 2011; Young et al., 2018). This involves the role of self, feeling connected with colleagues, and the personal environment (Ho et al., 2021). Ho et al. (2021) study on newly graduated Scottish nurses concluded by mentioning that transition experiences can directly impact retention by pointing out that 10 participants in this study either changed their jobs or were thinking of leaving nursing. A limitations of the above study was the time taken to collect data for this study (2 years) and some of the participants' experiences might have changed over that period. Hung et al. (2014) also highlighted that it is important to have a ‘sense of belongingness’ with colleagues and mental health settings. Additionally, a sense of belongingness contributed to the feeling of security and thereby smooth transition (Ho et al., 2021; Hung et al., 2014).

#### 4.1.2 Self‐efficacy and understanding of self

The term self‐efficacy is defined as the person's understanding and capacity to adapt behaviours to achieve goals (Bandura, 1997). Evidence suggests that there is a significant correlation between self‐efficacy and retention (Pelletier et al., 2019). The above quantitative time‐sequenced comparative study (Pelletier et al., 2019) of multiple groups of newly graduated mental health nurses used the Occupational coping self‐efficacy instrument to measure a person's self‐appraisals of capabilities to cope with environmental demands in mental health settings. The results of this study indicated that 11.7% of new nurses left their position within the first year and 2.9% left the profession within the second year (Pelletier et al., 2019). Most respondents indicated that factors that contributed were job dissatisfaction, understaffed units, patient load and acuity of the work environment (Pelletier et al., 2019). On the other hand, another study highlighted that resilience has also played a role in the retention of nurses (Cao et al., 2021). This study, which explained the effects of resilience on turnover intention in newly graduated nurses (Cao et al., 2021), postulated resilience as a personal factor that influenced retention. Higher levels of resilience positively influenced the process of transition. This study also indicated that transition shock could also affect the intention to leave nursing.

Nurses' understanding of self was also highlighted as a contributing factor to retention however portrayed as another factor that positively impacts the process of transition (Rahmani et al., 2019). Besides, self‐awareness also contributed to self‐confidence in the work environment. Self‐awareness is indicated as being aware of one's fears and anxiety while paying attention to the impacts of positive communication (Rahmani et al., 2019). To conclude, evidence asserts that positive experiences enhance a sense of belonging, self‐efficacy and self‐understanding contributing to a smooth transition and positive retention (Cao et al., 2021; Ho et al., 2021; Hung et al., 2014; Rahmani et al., 2019).

### 4.2 Professional factors

There were a number of professional factors identified and are described below:

#### 4.2.1 Influence of workplace environment

Many studies have emphasized the importance of organizational support in the transition and retention of nurses during the initial years of practice (Kaihlanen et al., 2020; Karlowicz & Ternus, 2009; Schwartz et al., 2011). The organizational factors include supportive colleagues and the workplace culture. Several studies aimed to explore the influence of the work environment on the experiences of new nurses and identified teams' dynamics and collegiality have crucial roles to play in transition. Most of the studies were qualitative, and two were using mixed‐method approaches (Cao et al., 2021; Kaihlanen et al., 2020; Schwartz et al., 2011; Wright et al., 2011; Karlowicz & Ternus, 2009;).

#### 4.2.2 Team dynamics and collegiality

The role of team members and their impact on transition is established in the literature (Wright et al., 2011). A qualitative study also analysed 10 semistructured interviews with new nurses and identified that the quality connection with the team members contributes to job satisfaction and transition (Wright et al., 2011). In congruent with the above, Schwartz et al.'s (2011) study also brought forth similar findings. Schwartz et al.'s (2011) study reported that support from the colleagues and clinical team made the nurses confident which has contributed to job satisfaction and integration. This study also pointed out that the social connection with the team members reduces the stress and anxiety of new nurses. Likewise, Schwartz et al. (2011) found that collaborating with team members could promote safety in mental health settings. Similarly, a study (Mabala et al., 2019) on the experiences of new nurses in South Africa indicated the importance of positive interaction within the team and the value of learning from colleagues. The team support will also assist the nurses to overcome anxiety related to the new mental health work environment (Mabala et al., 2019). Likewise, Karlowicz and Ternus (2009) also asserted the importance of team dynamics. This grounded theory research interviewed 14 newly qualified nurses and spotlighted that team and organization support directly impacts the retention of nurses during the initial years of practice (Karlowicz & Ternus, 2009). Additionally, it is suggested that confusion and ambiguity of roles and responsibilities in the work environment can negatively impact transition (Karlowicz & Ternus, 2009). Integration into the existing team resulted in job satisfaction, role clarity within the team and improved retention (Karlowicz & Ternus, 2009; Mabala et al., 2019).

#### 4.2.3 Workplace culture

Evidence from available literature calls attention to the influence of workplace culture on transition experiences and retention of nurses during their initial years of practice (Hazelton et al., 2011; Ho et al., 2021; Wright et al., 2011). Australian action research on new graduate mental health nurses (Hazelton et al., 2011) also stated that workplace environment, role modelling and supportive colleagues can positively impact transition experiences. Participants revealed their negative experiences while fitting into the work environment. Significantly, unfamiliar work environment, routine, judgemental attitudes of colleagues, lack of cooperation of team members and lack of support from experienced mental health nurses were associated with an increase in a sense of confusion. Likewise, a recent study (Ho et al., 2021) also stated that workplace factors such as lack of team support, and poor team morale could impact the experiences of new nurses. Participants in this study pointed out that transition shock was contributed by a negative workplace environment and influenced retention. Significantly, Hazelton et al. (2011) and Ho et al. (2021) identified that professional factors such as orientation, supervision, professional development and training can positively influence transition and retention during initial years of practice.

#### 4.2.4 Mentorship and support

Hazelton et al. (2011) studied newly graduated mental health nurses in public mental health services and found that mentorship and clinical rotations ensured a variety of learning experiences. Similarly, Karlowicz and Ternus (2009) also recommended that mentorship programmes for novice nurses assist in team‐building and improve retention. A formal mentorship programme ensures mentors' support in orienting to the new workplace, developing professional skills (Hazelton et al., 2011) and increasing job satisfaction (Karlowicz & Ternus, 2009). Karlowicz and Ternus (2009) also identified that organizational support was inevitable, and lack of support contributed to the nurses' decision to leave. Mabala et al. (2019) studied newly graduated nurses working in public mental health and identified that providing patient care responsibilities and throwing to the deep end without proper support and orientation could increase stress and turnover. This was particularly so during the initial weeks of mental health nursing (Hazelton et al., 2011; Mabala et al., 2019). An Iranian study on mental health nurses' perception of transition in mental health wards (Rahmani et al., 2019) also added that training and professional development during the initial years of practice could positively change the transition experiences.

Another research highlighted the strongest correlations between social support during the initial years of mental health nursing including positive behaviours and reflective learning (Pelletier et al., 2019). This study identified teamwork, mentoring and support from nursing and non‐nursing colleagues improved the new nurses' confidence in their knowledge and skills (Pelletier et al., 2019). The support provided for the new nurses made them confident in sharing their experiences with the team and reflective learning (Pelletier et al., 2019).

#### 4.2.5 Professional development

New nurses' transition and retention continue to remain challenging with various factors influencing the process. Professional development including transition programmes improves transition experiences and retention of nurses during the initial years of practice (Karlowicz & Ternus, 2009). Karlowicz and Ternus (2009), aimed to explore issues influencing psychiatric nurses' retention during the first year of practice. Participants of this study revealed factors including issues related to education and training, and lack of support had influenced their decision to leave within a year of practice. Another phenomenological study on graduate nurses from Taiwan (Hung et al., 2014) also reported that professional development including learning core competencies and the process of interaction with clients and families was important during the first year. Professional development including transition programmes and pre‐entry programmes indicated as an important point in the career before the transition to a nursing speciality (Karlowicz & Ternus, 2009).

## 5 DISCUSSION

This review aimed to identify the factors influencing the transition and retention of mental health nurses during the initial years of practice. Our findings indicated a paucity of research focusing specifically on factors influencing the transition and retention of mental health nurses during the initial years of practice. Two major factors with subfactors were elicited and were personal factors and professional factors that influenced the transition and retention of new mental health nurses. Personal factors such as self‐confidence and self‐efficacy were important attributes new nurses must possess during the initial years of their practice to remain in their field of choice. However, to develop these attributes, they must feel they belong in their working environment. Therefore, nurse managers and leaders need to develop and implement support strategies including mentoring and clinical supervision for these nurses. Mentoring and clinical supervision were identified as helpful tools for the health workforce, especially in mental health (Department of Health, 2018).

A study conducted by Kim and Shin (2020) examined the barriers and enablers for the transition of novice nurses in general nursing settings. The findings of the study indicated that self‐efficacy and self‐confidence were two significant personal factors influencing successful transition. Fears, over expectations, and emotional issues were highlighted as barriers to effective transition (Chiao et al., 2021; Cleary et al., 2009; Kim & Shin, 2020). The feeling of being accepted, respected and included in the workplace environment connected novice nurses with the workplace and therefore increased the chance for successful retention (Chamberlain et al., 2019; Guo et al., 2019). Chamberlain et al. (2019) asserted that ‘the sense of belonging was vital to retention’ (p. 9). These insights may guide nurse managers and leaders when involved in developing support guidelines for the successful transition experience in mental health settings. Many early career mental health nurses described that their self‐confidence increased within the first 6 months and was fastened when they felt supported (Ho et al., 2021; Hung et al., 2014). Adequate orientation, ongoing support and respect for novice nurses are essential in all their transition phases to build confidence. This highlights the need for a robust orientation and support plan for early career mental health nurses and is concurrent with the findings of this review.

Many nurses from various studies identified that team dynamics, collegiality and workplace culture had a crucial influence on the retention of novice nurses (Chamberlain et al., 2019; Hooper et al., 2016; Kim & Shin, 2020). An integrated review conducted by Hooper et al. (2016) highlighted that mental health graduates were highly likely to remain in the field when they were adequately supported and had positive experiences during their graduate year. The above findings are concurrent with the findings of this review, for example, team dynamics and the camaraderie of the team (Mabala et al., 2019) had significant roles in their transition experience as they felt part of the team. Moreover, social connection decreased their anxiety and stress and promoted a sense of safety, especially for those working in mental health settings (Schwartz et al., 2011). To summarize, although retention of early career mental health nurses is a known priority globally and in Australia (Government of Australia, 2020; Hayes et al., 2012; Mills et al., 2016), this review identified that there is a gap in research addressing this specific issue. Additionally, another aim of the review was to identify evidence‐based strategies for effective transition and retention. This was not completely achieved due to insufficient evidence from the existing literature.

## 6 LIMITATIONS OF THE REVIEW

We acknowledge that there might be a few limitations to this review due to the lack of formal quality assessment of selected studies and the likelihood of missing related literature due to the exclusion of grey literature. Most of the studies included in this review were completed before the COVID‐19 pandemic. During the pandemic, early career nurses experienced the stress of working in an unfamiliar environment and modified ways of working due to staff shortages and pandemic‐related restrictions. This situation might have impacted the experiences of early career nurses, which are not identified in this review.

## 7 CONCLUSION

This literature review highlighted several personal and professional factors that influence the transition and retention of mental health nurses during the early years of their practice. It has also indicated that a positive transition experience can contribute to the retention of nurses. However, there is a gap in the literature addressing effective strategies for positive transition experiences for early career nurses. Hence, future research is recommended to explore this area, especially during the initial years of mental health nursing career.

## 8 IMPLICATIONS FOR NURSING MANAGEMENT

Potential benefits of effective transition and support with the understanding of factors influencing transition and retention of early career mental health nurses will enhance staff morale, sustainability of the workforce and better patient outcomes. This review also has the potential of informing the organization and clinical leadership of factors influencing retention. This includes the importance of a supportive and collaborative work environment, especially during the initial years of nursing practice.

## CONFLICT OF INTEREST

The authors declared no potential conflicts of interest.

## ETHICS STATEMENT

No ethics approval was sought as this is a literature review paper.

## Supporting information


**Data S1.** Supporting Information

## Data Availability

Research data are not shared.
